# Applications of Topological Data Analysis in Oncology

**DOI:** 10.3389/frai.2021.659037

**Published:** 2021-04-13

**Authors:** Anuraag Bukkuri, Noemi Andor, Isabel K. Darcy

**Affiliations:** ^1^Department of Integrated Mathematical Oncology, Moffitt Cancer Center, Tampa, FL, United States; ^2^Department of Mathematics, University of Iowa, Iowa City, IA, United States

**Keywords:** topological data analysis, persistent homology, oncology, single cell analysis, imaging, clonal evolution, tumor heterogeneity

## Abstract

The emergence of the information age in the last few decades brought with it an explosion of biomedical data. But with great power comes great responsibility: there is now a pressing need for new data analysis algorithms to be developed to make sense of the data and transform this information into knowledge which can be directly translated into the clinic. Topological data analysis (TDA) provides a promising path forward: using tools from the mathematical field of algebraic topology, TDA provides a framework to extract insights into the often high-dimensional, incomplete, and noisy nature of biomedical data. Nowhere is this more evident than in the field of oncology, where patient-specific data is routinely presented to clinicians in a variety of forms, from imaging to single cell genomic sequencing. In this review, we focus on applications involving persistent homology, one of the main tools of TDA. We describe some recent successes of TDA in oncology, specifically in predicting treatment responses and prognosis, tumor segmentation and computer-aided diagnosis, disease classification, and cellular architecture determination. We also provide suggestions on avenues for future research including utilizing TDA to analyze cancer time-series data such as gene expression changes during pathogenesis, investigation of the relation between angiogenic vessel structure and treatment efficacy from imaging data, and experimental confirmation that geometric and topological connectivity implies functional connectivity in the context of cancer.

## 1. Introduction

With the advent of next-generation high-throughput sequencing (Roychowdhury et al., 2011; Reuter et al., 2015), improved medical imaging (Wang, 2016; Tahmassebi et al., 2018; Aiello et al., 2019), and an increased focus on personalized medicine (Dilsizian and Siegel, 2014; Gu and Taylor, 2014; Alyass et al., 2015; Suwinski et al., 2019), more data is being collected than ever before. Efficient data analysis techniques are critically needed to convert this data into meaningful, clinically translatable information. Topological data analysis (TDA) focuses on the shape of data, identifying both local and global structures at multiple scales. Consider a trivial example: suppose data points lie on a circle. The data points could represent customers' preferences or patient gene expression. In this case if a product or drug were targeted to the average person, the target would be the center of the circle and would thus miss the data set entirely. While this is a simple made-up example, it illustrates the importance of understanding the shape of data. TDA can be applied to high-dimensional and noisy data. While the output of TDA can be affected by incomplete data, it is still effective at distinguishing between data sets that have different shapes.

TDA has been successfully applied in a variety of medical contexts including to discover phenotype-biomarker associations in traumatic brain injury (Nielson et al., 2017), identify diagnostic factors for pulmonary embolism (Rucco et al., 2015), discriminate between healthy patients and those with diabetic retinopathy from retinal imaging (Garside et al., 2019), map human recombination at fine scales (Camara et al., 2016), identify novel pathological phenotypes of asthma (Siddiqui et al., 2018), and characterize the structure of chromatin conformation inside the nucleus (Emmett et al., 2016). In this review, we shall focus our attention on some recent applications of persistent homology, a main tool of TDA, to oncology. We specifically discuss treatment responses, clinical outcomes, disease classification, biomarker identification, and cellular architecture in cancer. We will also provide insights into possible future fruitful avenues of research, including analysis of time-series data to help with disease classification and identification of selection events, investigation of the relation between angiogenic vessel structure and treatment efficacy from imaging data, and experimental confirmation that geometric and topological connectivity implies functional connectivity in the context of cancer. Though we focus on persistent homology here, it is worth noting that there have been many notable successes of the application of other TDA methods, such as the *Mapper* algorithm (Singh et al., 2007). For example, *Mapper* was recently used to extract information from high-throughput microarray data and define a new subtype of breast cancer, c-MYB+, characterized by high c-MYB expression and low levels of innate inflammatory genes, with corresponding patients exhibiting 100% survival and no metastasis (Nicolau et al., 2007). In another study, *Mapper* was used to discover 38 new cancer-associated genes across tumor types, some of which were then confirmed to play a key role in tumorigenesis in mouse models (Rabadán et al., 2020). Before delving into the applications of persistent homology in cancer, we introduce some of the key mathematical underpinnings needed to understand these results.

## 2. What Is Persistent Homology?

**Figure 1:**
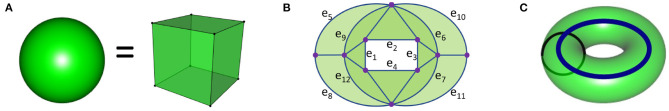
**(A)** The solid ball and solid cube are topologically equivalent and thus have the same homology. Their surface boundaries also have the same homology since these surfaces are topologically equivalent. The solid ball has one connected component and thus β_0_ = 1. The solid ball does not contain any voids, and thus β_*i*_ = 0 for all *i* > 0. The sphere, which is the boundary of the ball, has β_0_ = 1 since it is connected, and β_2_ = 1 since the 2-dimensional sphere bounds a void, while β_*i*_ = 0 for all other *i* since there are no lower or higher dimensional voids. For an *n* + 1-dimensional ball (for example, all points of distance less than or equal to 1 from the origin in *R*^*n*+1^), β_*i*_ = 0 for all *i* > 0 since it does not contain any voids. The *n*-dimensional sphere, which is the boundary of the *n* + 1-dimensional ball, has β_*i*_ = 1 for *i* = 0, *n* and β_*i*_ = 0 for all other *i*. Since the *n*-dimensional sphere contains a void, it is the *n*-dimensional object that generates β_*n*_. **(B)** A surface with boundary that is topologically equivalent to an annulus. The annulus is a 2-dimensional surface that has the same homology as a 1-dimensional circle. Since this object has one connected component, β_0_ = 1. We can use addition to represent a cycle. The cycle *e*_5_ + *e*_9_ + *e*_12_ + *e*_8_ = *e*_5_ + *e*_8_ + *e*_9_ + *e*_12_ is homologous to 0 since it bounds a surface (the light green waning crescent moon). Since all 1-dimensional cycles are either homologous to 0 or to (a multiple of) the rectangle cycle *e*_1_ + *e*_2_ + *e*_3_ + *e*_4_, β_1_ = 1. Since this object lives in the 2-dimensional plane, β_*i*_ = 0 for all *i* > 1. **(C)** The solid torus has β_*i*_ = 1 for *i* = 0, 1 and β_*i*_ = 0 for all other *i* while its boundary, the torus, has β_*i*_ = 1 for *i* = 0, 2, β_1_ = 2, and β_*i*_ = 0 for all other *i*. The thick blue cycle is a 1-dimensional homology generator for both the solid torus and its boundary. The thiner black cycle is a homologous to 0 in the solid torus as it bounds a meridinal disk, while this black circle is a homology generator in the torus which is not homologous to the blue circle. The torus surface generates the 2-dimensional homology.

The mathematical definition of homology/homologous is very precise and often differs from the English common usage. Homology uses algebra to detect topological shapes. Topology is sometimes called rubber sheet geometry as two objects are topologically equivalent to each other if one can be deformed into the other without tearing or puncturing the objects. For example, the spherical and cubical surfaces are topologically equivalent per Figure 1A. The sphere is topologically different from the 3-dimensional ball that the sphere bounds. Homology detects this difference by noting that the 2-dimensional spherical surface bounds a void while the 3-dimensional ball is solid and thus does not bound any voids.

To describe homology, we will first focus on two quantities: β_0_ = the number of connected components and β_1_ = the number of 1-dimensional holes (a circle that has not been filled in). One does not need to understand the algebra of homology in order to understand the basics of persistent homology, thus we will only briefly introduce some concepts for the interested reader. Two points are homologous if they are in the same connected component. Thus, β_0_ = 1 if the object is connected. To describe β_1_, we will focus on Figure 1B. We can use addition to represent topological objects. For example, the rectangle in Figure 1 is represented by the sum of edges: *e*_1_ + *e*_2_ + *e*_3_ + *e*_4_. Two 1-dimensional cycles are homologous to each other if they form the boundary of a surface. Thus, the rectangle is homologous to the cycle *e*_5_ + *e*_8_ + *e*_10_ + *e*_11_ since these two cycles bound the green surface. The cycles *e*_5_ + *e*_6_ + *e*_7_ + *e*_8_ and *e*_9_ + *e*_10_ + *e*_11_ + *e*_12_ are also homologous since they bound the light green surface consisting of two crescent moons. In fact all these cycles are homologous to the rectangle *e*_1_ + *e*_2_ + *e*_3_ + *e*_4_. One can see that this object contains many cycles, many of which are homologous to the rectangle (or a multiple of the rectangle, for example, $\overset{12}{\underset{i=5}{\sum }}e_{i}$ is homologous to $2\overset{4}{\underset{i=1}{\sum }}e_{i}$). A 1-dimensional cycle is homologous to 0 if it bounds a surface. Thus the cycles *e*_5_ + *e*_9_ + *e*_12_ + *e*_8_ and *e*_6_ + *e*_7_ + *e*_11_ + *e*_10_ are both homologous to 0 since they each form the boundary of a surface (the two crescent moons, waning or waxing, respectively). Since each of the cycles in this figure are homologous to 0 or to a multiple of the rectangle, its homology is generated by a single cycle (for example, the rectangle) and thus β_1_ = 1.

The intuitive definition of homology is that β_*n*_ equals the number of *n*−dimensional holes^1^. Per the Figure 1 caption, homology can be used to distinguish the following objects from each other: solid ball, sphere, higher dimensional balls and spheres, solid torus, and torus. Homology cannot distinguish all objects that are topologically different. For example,the 1-dimensional circle, the 2-dimensional surface in Figure 1B, and the 3-dimensional solid torus (Figure 1C) all have the same homology. For more on the mathematical definition of homology (please see Munkres, 1984; Hatcher, 2002; Ghrist, 2014).

**Figure 2:**
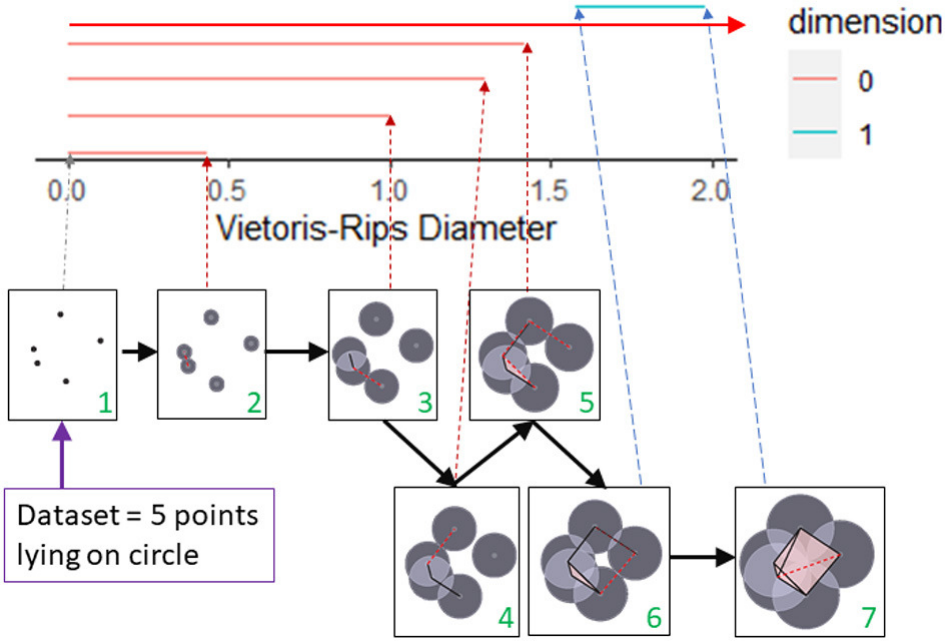
A barcode captures topological features in a dataset at multiple scales. The topology of a dataset at a fixed scale is determined by joining pairs of data points with an edge if the distances between the pair of points is less than the fixed scale. If three edges form a triangle, then the triangle is filled in. This process is shown in the seven boxes as the scale for joining vertices increases from box 1 to box 7. The corresponding barcode is shown at the top of the figure. The persistence of a feature over multiple scales determines the length of the bar corresponding to that feature. The number of components (β_0_) that exist at a particular scale is represented by the number of red bars that exists at the corresponding Rips diameter. The creation of the 1-dimensional cycle in box 6 is represented by the birth of the blue bar. The blue bar dies when this cycle is filled in with triangles (box 7). This figure was created by modifying the output of the R package TDAstat (Wadhwa et al., 2018) and latex code written by Catalina Betancourt.

We will illustrate with an elementary example how persistent homology can detect shape at multiple scales by noting the birth and death of topological features. Our dataset will consist of 5 points from a circle as shown in Figure 2. To detect the circle, we need to connect these points in some manner. For example, we could connect all points whose distance is less than some fixed ϵ. If one can visualize the data set, then the choice of ϵ may be clear. But more often, there is no obvious choice, so instead we analyze the data at multiple scales using persistent homology. The first box in Figure 2 shows the five data points. At this stage, we have five components, one for each data point (β_0_ = 5). These components are represented by the five red lines in the top part of this figure. These five red lines along with the blue segment is called the barcode for the data set. The barcode keeps track of the number of components (red bars) and number of 1-dimensional holes (blue bar) as the threshold for connecting data points increases. We can visualize the increasing threshold (or proximity parameter) by growing balls around each data point and connecting pairs of points as soon as their respective balls intersect. Thus, in the second box, an edge joins the two closest points, reducing the number of connected components by one. Thus, one bar ends (dies), and only 4 bars (β_0_ = 4) continue past this threshold. Observe that every time an edge joins two components, a bar dies (and β_0_ reduces by one). In the timepoint just before 1.5 (box labeled 5), two edges are added. One connects two components, but the third forms a triangle with two previously created edges. These three edges surround a small hole, but we fill in this hole (shaded in pink) as we only want to detect large holes. We are forming a Rips complex where whenever a triangle is formed, it is immediately filled in and thus triangles do not contribute to β_1_. In the timepoint after 1.5 (box labeled 6), a cycle containing four edges is formed. This is indicated in the barcode by the start (birth) of the blue bar. As more edges are added, eventually this region is divided into two triangles and the blue bar dies at timepoint close to 2 (corresponding to box labeled 7). Note we have one infinitely long bar (top red bar with arrow) since after time 1.5 we have one connected component.

To summarize, this example of a TDA pipeline consists of taking a dataset, creating a sequence of Rips complexes, and outputting a barcode (Edelsbrunner et al., 2002; Carlsson et al., 2005; Zomorodian and Carlsson, 2005). A Rips complex is a generalization of a graph. While in our example we only looked at adding edges and triangles, we can also add higher dimensional simplices. A *n*-simplex in a Rips complex is a collection of *n* + 1 points where each pair of points is connected by an edge. Thus an edge is a 1-simplex, a triangle is a 2-simplex, and a tetrahedron is a 3-simplex. In our circle example, when all pairs of the 5 points are connected by edges, we add a 4-simplex even though the data set lives in 2-dimensions. The existence of an *n*-simplex means that (all pairs of) *n* + 1 points are close together according to a given threshold. The Rips complex is also called a clique complex, the latter term coming from graph theory where a clique is a graph where every pair of vertices is connected. Thus, our simplices correspond to clique subgraphs. Other names for Rips complex include Vietoris-Rips complex and flag complex.

There are other ways to form a simplicial complex from data. For the Rips complex, an *n*-simplex is formed at threshold *r* when all pairs of *n* + 1 points are of distance less than *r* (so that each pair of points is connected by an edge). This is equivalent to requiring every pair of balls of radius *r* centered around the *n* + 1 points to intersect. If we require the intersection of all these balls to be nonempty in order to form an *n*-simplex, we instead form the Čech complex. Thus, to form a 2-simplex (triangle), the Rips complex only requires non-empty pairwise intersection of three balls while the Čech complex requires the intersection of all three balls to be nonempty. Thus, the Čech complex is similar to the Rips complex, but an *n*-simplex is formed at a slightly larger threshold in the Čech complex. Under certain conditions, the Čech complex is guaranteed to have the same homology as the union of all balls of radius *r* centered around data points (Hatcher, 2002). But the Rips complex has much smaller computer memory requirements as only the edges need to be stored to determine the Rips complex, and thus the Rips complex is normally used when calculating persistent homology. A very different TDA technique called Mapper uses a completely different method to create a simplicial complex from data (Singh et al., 2007). For Mapper, each vertex represents a cluster of data points. If *n* + 1 of these clusters have a common intersection, then an *n*-simplex is formed. Mapper can be used to reduce the size of a data set and to visualize it.

The example in Figure 2 focused on β_0_ and β_1_. For data that lives in a higher dimensional space, we can similarly calculate β_*n*_ = the number of n-dimensional holes. For example, β_2_ = 1 for both the sphere and torus as these are 2-dimensional surfaces that bound voids in space. For more details regarding persistent homology and barcodes (please see Ghrist, 2008; Carlsson, 2009; Edelsbrunner and Harer, 2010; Otter et al., 2017).

In order to use persistent homology in machine learning, we need a distance between barcodes. We first convert barcodes to persistence diagrams as described in the next section and use these diagrams to define a distance between barcodes. In this section, we show how persistent homology is stable with respect to noise: small perturbations in the data have only a small effect on the barcode (Cohen-Steiner et al., 2007). In section 2.2, we discuss the advantages/disadvantages of persistent homology with regard to how it handles noise, incomplete data, and computational complexity. In section 2.3, we discuss one method (persistent images) of converting a persistence diagram into a vector that can be used in machine learning. We also give references to many other methods for using persistent homology in machine learning.

While we have discussed the basic method for converting Euclidean data into barcodes, there are a number of other methods for obtaining barcodes from data. All one needs is a method to determine when to add an edge between pairs of data points. Thus, the data do not need to live in Euclidean space. We also assumed that small holes correspond to noise, but there are applications where the point of using persistent homology is to detect small holes (Bendich et al., 2016). We also had only one infinite bar corresponding to the one connected component we obtained when all our data points were connected by edges. If one is working with Euclidean data, eventually all holes will be filed in and thus eventually a Rips complex with only one component and no holes will be formed. But in other applications, holes may persist forever, resulting in infinite bars. One can also obtain additional information by looking at the group structure of the filtered homology groups, and prove stability properties using interleaving distance (Bauer and Lesnick, 2014; Bubenik and Scott, 2014; Oudot, 2015; Chazal et al., 2016).

### 2.1. Persistence Diagrams and Stability

**Figure 3:**
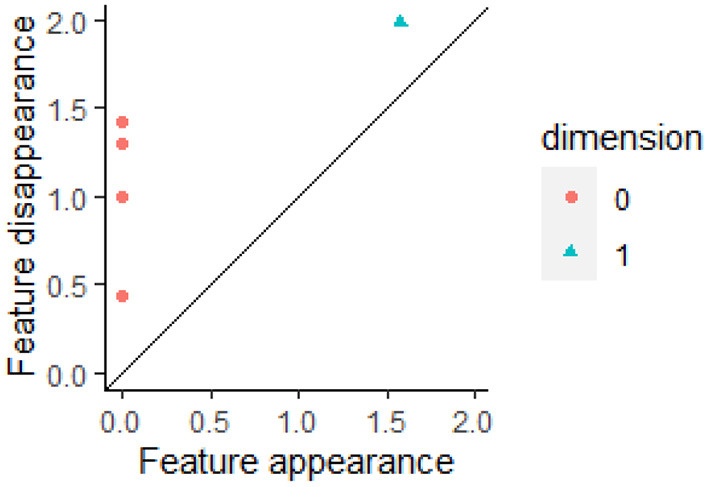
A barcode can be converted into a persistent diagram. Each bar with finite length in a barcode is represented by a point in the persistent diagram. If a bar is born at time *b* and dies at time *d*, then the bar is represented by the point (*b, d*). In Figure 2, there are four finite red bars plus one infinite red bar. These bars are all born at time 0. In the persistent diagram, the four finite red bars are represented by the four red points all of which have *b* = 0. The one blue bar in Figure 2 is represented by the blue triangle in this persistent diagram. This figure was created using the R package TDAstat (Wadhwa et al., 2018).

While barcodes are useful for visualizing changes in homology, barcodes are generally converted into persistence diagrams for statistical and machine learning analysis (Edelsbrunner et al., 2002; Mileyko et al., 2011). The start of a bar represents the birth of a cycle while the end represents its death. The plot of the points (birth time, death time) in 2-dimensional space is called the persistent diagram (PD). The persistent diagram corresponding to the barcode in Figure 2 is shown in Figure 3. A persistence diagram also includes the diagonal as shown in this figure as the diagonal is used when computing distances between PDs. A PD can be a multiset if multiple bars have the same birth time *b* and death time *d*, so that the point (*b, d*) occurs multiple times in the PD.

**Figure 4:**
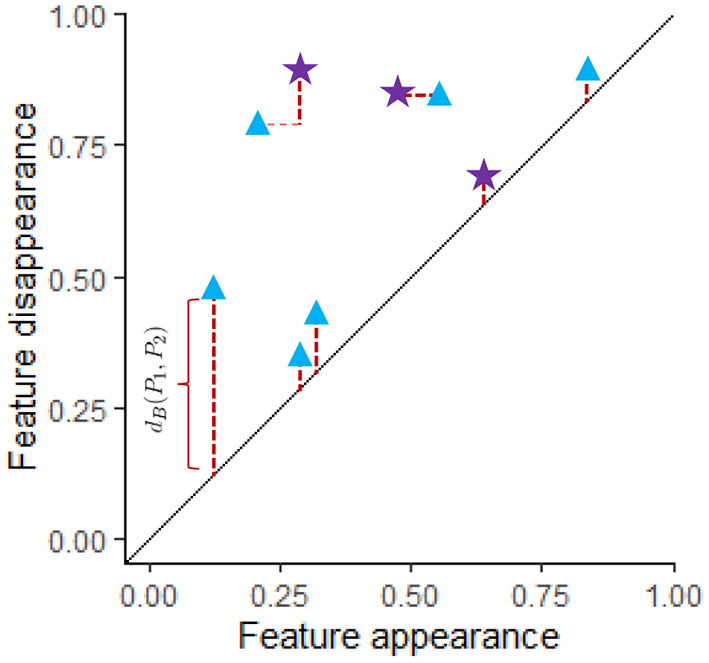
Two persistence diagram, *P*_1_ and *P*_2_, are shown for a single dimension (for example, β_1_). The blue triangles correspond to *P*_1_ while purple stars are used for *P*_2_. Both persistence diagrams include the diagonal. A matching between *P*_1_ and *P*_2_ is shown where the red dotted lines indicate features that have been matched where some of the features are matched to the diagonal. The length of the thicker dark red dotted lines indicate the distance between matched features. The distance between a feature and the diagonal is the persistence of the feature, *d* − *b*, where *b* = birth time and *d* = death time of that feature. If feature (*b, d*) is matched with feature (β, δ), then the distance between these features is max(|*b* − β|, |*d* − δ|). Since the best matching is shown, *d*_*B*_(*P*_1_, *P*_2_) equals the length of the longest of the thick dark red dotted lines. Any other matching would have a matched pair of features with larger distance.

The formula for the bottleneck distance for a fixed β_*i*_ between two persistence diagrams, *P*_1_ and *P*_2_, is $d_{B}\left(P_{1},P_{2}\right):=\underset{\gamma :P_{1}\to P_{2}}{\inf}\;\underset{x\in P_{1}}{\sup}∥x-\gamma \left(x\right){∥}_{\infty }$. To compute this distance we first create a matching γ between these diagrams for the fixed β_*i*_ as shown in Figure 4. In this figure the blue triangles represent features with the fixed β_*i*_ from one data set while the purple stars represent features from a different data set for the same β_*i*_. A matching γ:*P*_1_ → *P*_2_ is a bijective function from *P*_1_ to *P*_2_ where both persistence diagrams include the diagonal. Features that are close to the diagonal get matched to the diagonal unless they are closer to another feature that does not have a better matching than to the diagonal. If *x* = (*b, d*) ∈ *P*_1_ is matched to the point (β, δ), then the distance between these features is ‖*x* − γ(*x*)‖_∞_ = max(|*b* − β|, |*d* − δ|). To find the distance for a particular matching γ, we calculate ${\text{sup}}_{x\in P_{1}}∥x-\gamma \left(x\right){∥}_{\infty }=$ the largest distance between a point *x* in *P*_1_ and its match γ(*x*) in *P*_2_. The bottleneck distance is obtained by taking the infimum of this distance over all possible matchings. In Figure 4, red dotted lines indicate best matches between features from *P*_1_ and *P*_2_.

If *P*_1_ is the PD for the data set *X* and *P*_2_ is the PD for the data set *Y*, the stability theorem states that *d*_*B*_(*P*_1_, *P*_2_) ≤ *d*_*H*_(*X, Y*) = inf{ε ≥ 0; *X* ⊆ *Y*_ε_ and *Y* ⊆ *X*_ε_} where $X_{\varepsilon }:\;=\underset{x\in X}{\cup }\left\{z\in M;d(z,x)\leq \varepsilon \right\}$ (Cohen-Steiner et al., 2007). In other words, if each data point is perturbed by at most a distance ϵ, then the persistence of a feature will change by at most 2ϵ since the birth and death times can change by at most ϵ. Features with persistence <2ϵ may disappear, while new features with persistence less than 2ϵ may be created.

### 2.2. Benefits and Limitations of Persistent Homology

That persistent homology is stable with respect to noise is, of course, a major advantage. But any method that uses Euclidean distance is affected by the curse of dimensionality due to the effect of noise on distance. For example, suppose a data point should be at the origin, but due to noise, each coordinate is perturbed by 0.01 units, then the point which should be at the origin is now $\Sigma_{i=1}^{n}\left(0.01^{2}\right)$ units away from the origin if the data lives in ℝ^*n*^. Thus, for example if *n* = 10, 000, then the data point is perturbed by a distance $\Sigma_{i=1}^{10,000}\left(0.01^{2}\right)=$ 1. While the change in persistent homology is bounded by the distance between the original data set and the perturbed data set, the latter can be quite large, depending on the amount of noise and the dimension of the dataset. Thus, performing PCA or t-SNE or other dimension reduction technique first may lead to stronger results.

In order to recover the shape of an object, one must have sufficient coverage. Some holes detected by persistent homology may be due to incomplete data. If these are small, then they only result in short bars which may be considered noise. But in high dimensional spaces, one has many degrees of freedom, so even recovering the shape of simple objects in high dimensions can be impossible as obtaining a sufficient number of data points may not be feasible. However, differences between data sets may still be detected even if coverage is lacking. For example, one may have insufficient coverage to recover the topology of a torus if one uniformly under-samples data points from a torus. However, the resulting barcode will likely be very different than the barcode obtained from uniformly under-sampling points from a sphere. Also, coverage can be less of an issue if you have some information regarding the shape of the data such as periodicity (for example, Dequeant et al., 2008). Thus, in practice, topological data analysis has proven to be quite robust. For more on complexity and topological inference (see Weinberger, 2014).

Due to computational complexity, most analysis using TDA restricts to the use of β_*i*_ for *i* ≤ 4. Often only β_0_ and β_1_ are used, but faster algorithms such as Ripser (Bauer, 2019) are becoming available. To calculate persistent homology of a point cloud, one first needs to create simplicial complexes. The number of simplices grows rapidly with the number of data points as well as the homology dimension (not the dimension of the data set, but the dimension of the holes one wishes to detect—in order to calculate β_*i*_, one needs *i*-dimensional and *i* + 1 dimensional simplices). The TDA pipeline also requires the computation of distances between data points. The dimension in which the data lives can affect this step, but after distances are calculated, it is the shape of the data that can have the largest effect, sometimes even larger than the number of data points as there are several algorithms that can greatly simplify the simplicial complex (Zomorodian, 2010; Mischaikow and Nanda, 2013; Wilkerson et al., 2014; Boissonnat and Pritam, 2020). The effectiveness of these simplification algorithms depends on both the topology and geometry of the data set. For example, suppose one takes *n* data points equally spaced on a straight line. The topology of the line is the same as the topology of a point. Thus, to calculate the homology of the line, one can remove all simplices except for a single vertex. For more on computational complexity of persistent homology (see Otter et al., 2017).

If all the data points enter at time 0, the β_0_ bars all start at time 0. Thus the barcode for β_0_ can be created from a single linkage hierarchical clustering dendrogram as the merge heights of the dendrogram become the lengths of the β_0_ bars. Hence the β_0_ barcode contains less information than a single linkage hierarchical clustering dendrogram. However, there are applications where the data points enter at different times such as time series data. Thus, the β_0_ barcode can be applied to a wider variety of applications than standard clustering techniques. Clustering also cannot capture holes and voids; the higher dimensional barcodes capture structure that other methods such as clustering miss.

### 2.3. Persistent Homology and Machine Learning

**Figure 5:**
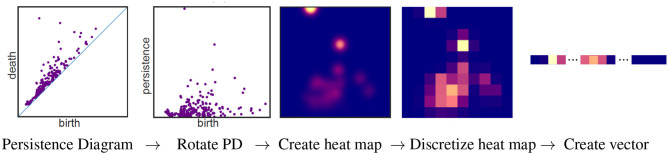
Pipeline for vectorizing a persistent diagram using persistent images. This figure is a modification of Figure 1 from Adams et al. (2017) which is licensed under CC BY 4.0.

The barcode can be used as a topological signature to identify structure in data. While homology is built to detect topology and not geometry, persistent homology can be implemented in a variety of ways to distinguish geometrical shapes (e.g., Turner et al., 2014; Li et al., 2018; Bubenik et al., 2020). Machine learning can be applied to a collection of persistent diagrams to distinguish between data sets with different structures. Many machine learning algorithms take a vector as input. There are many ways to create a vector from persistent homology. A pipeline to create a vector using persistence images (Adams et al., 2017) is illustrated in Figure 5. A persistent diagram is first rotated by 45° so that the diagonal becomes the horizontal axis (2nd panel of Figure 5). Thus the horizontal axis represents the birth time, while the vertical axis represents persistence = death - birth. A heat map is then created using a Gaussian distribution (or other weight function) about each point (3rd panel). The height of the Gaussian distribution is indicated with color in the heat map and is dependent on the persistence of the feature. Points closest to the diagonal are considered to be the result of noise and are thus given no intensity. Hence the bottom of the heat map will always have the color corresponding to zero intensity, in this case blue. In other words, points close to the diagonal have no effect on the heat map. Observe that the point furthest from the diagonal in the first panel corresponds to a feature with the largest persistence per second panel. Thus, in the heat map in the 3rd panel, the color at this point is given the highest intensity (yellow). As shown in the fourth panel, the heat map is discretized by partitioning the heat map into *n* × *n* squares where the color of each square corresponds to the average value of the corresponding square in the heat map. In the discretized heat map (4th panel), the yellowish region from the 3rd panel corresponding to the most persistent feature is partitioned between two squares with the yellow square in the top row of this heat map containing a larger portion than the pinkish square next to it in the same row. In the final panel, an *n*^2^-dimensional vector is created by concatenating the rows of the discretized heat map.

Other methods for using persistent homology in machine learning include persistent landscapes (Bubenik, 2015, 2020), persistent curves (Chung and Lawson, 2019), and kernel functions (for example, Reininghaus et al., 2015; Kusano et al., 2016; Carrière et al., 2017; Chazal et al., 2017).

## 3. Treatment Responses and Prognosis

What impedes the success of cancer therapies is often the coexistence of therapy resistant cells along with therapy sensitive tumor cell populations. When administered separately, all currently adopted therapeutic strategies—ranging from cytotoxic chemotherapies to molecular targeted therapies—impose a dramatic, yet homogeneous selective pressure on an often heterogeneous group of tumor cells. Despite varying resistance mechanisms contingent upon therapy-type and tumor composition, every therapeutic intervention inevitably selects for resistant cells, which expand and become the dominant cell type of recurrent tumors, that cease to respond to therapy (Maley and Reid, 2005; Aparicio and Caldas, 2013; Bukkuri, 2020). The increased resolution on the clonal architecture of intermixed tumor cell populations that has just now become available calls for prognostic and therapeutic benefits. High intra-tumor diversity in pre-malignant lesions has been shown to predict progression to malignant growths and poor outcome (Maley et al., 2006; Laurie et al., 2012). The therapeutic significance of intratumoral heterogeneity (ITH) is exemplified in a recent study that measured genetic and transcriptional diversity of breast cancer tumors before and after therapy based on four genetic markers and two transcriptional markers. The study provided proof-of-principle that therapy-induced phenotypic changes can be predicted based on the characterization of coexisting tumor subpopulations (Almendro et al., 2014). Another recent study used RNA interference to model heterogeneous tumors and tested the efficacy of predicted drug combinations in eliminating coexisting tumor subpopulations (Zhao et al., 2014). Their findings suggest that the most effective drug combination for a given tumor cannot be achieved by targeting the predominant subpopulation alone, but requires detailed characterization of the genetic makeup of branched subpopulations and their contribution to the tumor bulk.

Techniques from computational homology have been used to develop a new algorithm to characterize comparative genomic hybridization (CGH) profiles and identify the frequency of cancer recurrence in early stage breast cancer patients through identification of recurrent copy number aberrations (CNAs) in cancer (DeWoskin et al., 2010), which serve as markers of genomic instability and thus cancer prognosis (Hanahan and Weinberg, 2000; Han et al., 2006). Specifically, the method uses a sliding window algorithm to associate a set of point clouds to each array CGH. Different window sizes allow one to analyze the data at various scales by considering different dimensional point clouds. Then, persistent homology is applied to these point clouds for classification. It was found, in accordance with prior results (Climent et al., 2007), that the Betti numbers of the zero dimensional homology groups (β_0_) can distinguish between recurrent and non-recurrent groups in patients who did not receive anthracycline-based chemotherapy after surgery but not in patients who were treated with anthracycline. Note that, in this approach, no segmentation of the data was required.

In another study, a novel statistic called the smooth Euler characteristic transform (SECT), which allows shape information to be integrated into traditional statistical models, was developed and applied to predict disease free survival in glioblastoma multiforme (GBM) based on tumor shape from post-contrast T1 axial magnetic resonance imaging (MRI) (Crawford et al., 2020). SECT is a variation of the persistent homology transform (PHT) introduced in Turner et al. (2014) that was created to overcome the difficulties in integration with traditional statistical models. Specifically, the output of SECT is a collection of smooth vectors, while the output of PHT is a collection of persistence diagrams (Edelsbrunner et al., 2002), thus having a complicated representation and geometry which does not lend itself easily into integration with statistical models. In the GBM application, the statistical model used was a Bayesian linear mixed model (BLMM) (Ishwaran and Rao, 2005; Guan and Stephens, 2011; Zhou et al., 2013). When this topological approach was applied to the GBM MRI data, it was found to outperform gene expression, volumetric, and morphological summaries in predicting disease free survival.

Clinically, there is a great importance in the identification of biomarkers which can serve as predictors for metastasis and patient prognosis in cancer. To this end, researchers have recently used persistent homology techniques, in an exploratory data analysis fashion, to identify biologically meaningful geometric properties of single cell data (Lockwood and Krishnamoorthy, 2015). In this method, data was first transposed and analyzed in its dual space with each gene represented in a much lower dimensional sample space, thus circumventing the problem of high dimensionality that is typical of single cell data. A small set of genes (120–200) were then selected as landmarks (De Silva and Carlsson, 2004) and a family of nested simplicial complexes was constructed, indexed by a proximity parameter. Unlike many other methods which focus on the analysis of zero dimensional homology groups (DeWoskin et al., 2010; Nicolau et al., 2011), thus performing analyses which are topologically equivalent to clustering, this study focused their efforts on identifying loops of one dimensional homology groups which persist over a large range of values of the proximity parameter, hypothesizing that connections around holes imply nontrivial interactions among genes and biological functions which could have implications for tumorigenesis. Repeating this process for various landmarks, features which remain stable over large ranges of both the proximity parameter and number of landmarks could be detected. Applying these techniques to five different cancer data sets from brain, breast, ovarian, and acute myeloid leukemia cancers, many members of the significant loops in the one dimensional homology groups that were found have been previously shown to be accurate biomarkers for cancer biogenesis, while others serve as potential new markers which have yet to be experimentally validated.

## 4. Tumor Segmentation and Computer-Aided Diagnosis

Computerized methods can efficiently and effectively identify quantitative image features that are otherwise difficult to spot by manual inspection (Yu et al., 2016). Quantitative morphological features extracted from H&E stained slides, such as Zernike shape features, have been shown to predict survival in lung adeno- and squamous cell carcinoma (Yu et al., 2016). Recent advances in next-generation sequencing technologies gave rise to a plethora of approaches that quantify and characterize the genotypic diversity within a given tumor. Evidence supporting a quantitative relation between genotypic and morphological ITH followed. A quantitative image analysis approach that complements genomic profiling with geographical information was developed (Yuan et al., 2012; Andor et al., 2016). Furthermore, the authors characterized cellular heterogeneity by distinguishing between well-defined cell-populations (stromal cells, lymphocytes, cancer cells). However, so far qualitative details of how this diversity in morphology is structured (i.e., how many subpopulations are present and what their geographical boundaries are on the H&E slide) are unknown.

As a step toward a computer-aided cancer diagnosis system, persistent homology has been used to develop an automated tumor segmentation approach for Hematoxylin & Eosin (H&E) stained colorectal cancer histology whole slide images (WSI) (Qaiser et al., 2016). The authors exploit the fact that nuclei in tumor regions have atypical characteristics such as non-uniform chromatin texture, irregularity in shape and size, and clustering of nuclei, and use persistent homology profiles to characterize the degree of connectivity among nuclei and to classify cancerous regions based on this information. Specifically, once a WSI has been obtained, it is first divided into patches, each of which has a persistent homology profile. Given two patches, the symmetrized Kullback-Leibler divergence (KLD) can be computed between the respective persistent homology profiles, which serves as a metric for interpatch distance. Then an input patch is classified as cancerous or non-cancerous by a kNN classifier, based on KLD distances between its persistent homology patch and those of each representative patches. These exemplar patches are chosen by training a CNN and selecting patches whose activation during training is large (separately for cancerous and non-cancerous classes). The benefit of this approach over previous approaches is that only the subset of highly activated patches from the convolutional layers are used as exemplars rather than the set of all patches in the training data. This method was compared against standard CNN and HyMaP (Khan et al., 2013) approaches on 74 H&E stained WSIs of colorectal cancers; in addition to being computationally less expensive than the other two methods, it was also shown to have better precision and segmentation accuracy.

Another example of tumor segmentation and algorithmic diagnosis is a recent study which aimed to segment a diseased area of skin and classify the type of skin lesion into one of seven classes in a given dermatoscopic image (Tschandl et al., 2018) using persistent homology (Chung et al., 2018). Like the colorectal image segmentation study (Qaiser et al., 2016), the segmentation algorithm used is a concept similar to persistent homology (Edelsbrunner et al., 2002). Linear support vector machines (SVMs) were used for classification on the persistence statistics (Chung et al., 2018) and persistence curves (Chung and Lawson, 2019) were derived from persistence diagrams. Specifically, given an image, a segmentation algorithm was first implemented to obtain an image mask: a binary image in which each pixel is colored either white (if it part of the healthy skin) or black (if it is part of a lesion). Once the mask was applied to the original image, the RGB color space is transformed into an RGB, HSV, or XYZ color space and each channel was extracted. Persistent homology software was then used to compute persistence diagrams for each channel; from each diagram, persistence statistics and curves were computed as features. Finally, a multi-class SVM was used to classify the input into one of the seven types of skin lesions. When this approach was applied to a validation set of 5,000 images, the highest resulting accuracy scores were 65.6, 66, and 67.2%.

Similar persistent homology techniques were used to classify H&E stained stage T3 and stage T4 colorectal adenocarcinomas images as benign or malignant (Chittajallu et al., 2018). To do this, given an image, it was first color normalized (Reinhard et al., 2001) and the nuclear stain and minimum cross entropy thresholding (Li and Tam, 1998) for nuclear foreground segmentation were extracted using an unsupervised color deconvolution method (Macenko et al., 2009). Then, a fast difference-of-Gaussian implementation of the scale-adaptive Laplacian-of-Gaussian filter of Al-Kofahi et al. (2010) was performed to detect nuclei centroids. Then, by considering the set of nuclei centroids as a point cloud, the persistence diagram of its Vietoris-Rips filtration for the one dimensional homology groups (loops) was computed using a fast multiscale approach (Doyle et al., 2008). Then, persistence landscape (Bubenik, 2015) and image (Adams et al., 2017) representations were computed and used as features to characterize loops formed by glandular epithelial cell nuclei. Then given training images with benign/malignant labels, a random forest classifier was trained using these topological features. PCA was used to reduce the dimensionality of each feature group so as to preserve 99% of the variance. Hyperparameter optimization was also performed via cross-validation using a tree-structured parzen estimator (Bergstra et al., 2011). When this method was applied to testing data consisting of 80 images, an accuracy of 85%, AUC of 0.85, precision of 78%, and recall of 95% was obtained, an improvement over the traditional cell graph property approach in all areas (Doyle et al., 2008).

## 5. Disease Classification

Cancers of unknown primary represent 3–5% of all cancer cases, whereby physicians find one or multiple metastases but fail to locate the primary tumor. Pathologic evaluation of a metastatic biopsy often does not provide a definitive answer. Molecular data ranging from gene expression to somatic mutations have been shown to significantly aid classification of metastatic biopsies to their corresponding primary tumor site (Ferracin et al., 2011; Marquard et al., 2015; Vikeså et al., 2015; Moran et al., 2016; Søndergaard et al., 2017).

One study used persistent homology on 150 non-contrast-enhanced fat-suppressed 3D T1-weighted magnetic resonance (MR) images to classify hepatic tumors into three classes: hepatocellular carcinomas (HCC), metastatic tumors (MT), and hepatic hemangiomas (HH) (Oyama et al., 2019). To do this, for each image, a 3D region of interest (ROI) in the shape of a rectangular solid enclosing the entire lesion was created by an experienced radiologist. Then, gray-scale values of the voxels in each ROI were normalized and persistence diagrams were created for dimensions 0, 1, and 2 using HomCloud (Kimura et al., 2018; Obayashi and Hiraoka, 2018). These diagrams were vectorized into persistence images (Adams et al., 2015). Feature vectors were then obtained from these images and inputted into logistic regression with an elastic net penalty and extreme gradient boosting machine learning models for classification. The results from classification showed that dimension 1 persistence images had the highest accuracy rates: 85% for classifying HCC and MT, 84% for HCC and HH, and 74% for HH and MT.

An alternative method to accurately classify tumor subtypes is through the use of high throughput genomics (Nutt et al., 2003; Freije et al., 2004). Aiming to produce more robust algorithms than traditional classification methods, given gene expression profile data, researchers used statistical invariants and persistent homology to identify core patient groups associated with the classical, mesenchymal, and proneural subtypes of GBM and a compact set of genes most useful for this partitioning (Seemann et al., 2012). To do this, a sufficient, but compact, panel of genes to be used for clustering was predetermined using non-dimensionalized standard deviation (to ensure bimodality of gene expression distribution across patient samples; Phillips et al., 2006; Verhaak et al., 2010) and persistent homology (to find groups of genes whose expression levels change coherently among patient samples; Carlsson, 2009; Horak et al., 2009). Then, a hierarchical partitioning of patient samples based on gene expression levels is performed using persistent homology; specifically, samples are repeatedly bisected until further partitioning is not possible, thus obtaining the number of clusters that exists and some notion of genetic proximity of the clusters. Each bisection was implemented using 30 genes. A predictive model was then implemented to assign cancer subtypes to each cluster. Applying this approach to the 20 GBM test samples, fifteen predictions were in accordance with results from standard clustering calculations (Verhaak et al., 2010), five of which were unassigned by both algorithms. Of the remaining five samples, four were classified as “neural” by the clustering algorithm, but were unassigned by this approach since the neural group was not found in a single cluster.

Another example of the use of persistent and computational homology on gene expression data is in Arsuaga et al. (2012), whereby, upon application to a breast cancer gene expression dataset, the algorithm was able to distinguish among most breast cancer subtypes. This paper extended the work of DeWoskin et al. (2010) to gene expression data, under the assumption that gene expression is a measure of the underlying copy number changes (Neve et al., 2006; Horlings et al., 2010). Before applying the sliding window algorithm developed in DeWoskin et al. (2010) to gene expression data, theoretical work was done to show that under idealized conditions, the point cloud defined by the algorithm is a good representation of the original data. Hence, analysis of the point cloud is applicable to the original data set. This was done using Taken's embedding theorem, an extension of Whitney's embedding theorem to dynamical systems theory, and a circularization technique. To apply the sliding window algorithm to gene expression data, instead of pre-selecting differentially expressed genes like traditional clustering algorithms, all genes were ordered by their location in the genomes. Then, the sliding window algorithm was applied to generate point clouds, upon which topological and statistical analysis was performed. It was shown that when only β_0_ was used, the algorithm could distinguish between less aggressive subtypes, like normal and luminal-A, and more aggressive ones, such as luminal B, basal-like, and Her2. It was also noted that the algorithm could not distinguish luminal B from Her2 and basal-like, implying the close similarities among these subtypes. Thus, it was noted that breast cancer subtypes can not only be classified by specific sets of genes, but also by certain global relationships among all genes.

## 6. Cellular Architecture

Imaging is an essential part of cancer clinical protocols, providing physicians with morphological, structural, and metabolic information about patient tumors, thereby assisting in clinical decision making and treatment planning (Fass, 2008). The development of new image segmentation tools (Zhang et al., 2001; Hong and Brady, 2003; Xiaohua et al., 2004) and quantitative multiplex immunofluorescence (Stack et al., 2014; Dimitriou et al., 2019; Abousamra et al., 2020) have set the stage for topological data analysis and persistent homology techniques to be harnessed for interpretation of high-dimensional information in histopathological imaging data.

One example of this is using persistent homology techniques to investigate architectural characteristics of cellular organization and nuclear arrangements from microarray tissue samples to distinguish among genetically derived breast cancer subtypes (Basal, Luminal A, Luminal B, and HER2; Singh et al., 2014). This was done through distinct topological characterizations such as nuclear connectivity (generators of zero dimensional homology groups) and loops (generators of one dimensional homology groups) based on Vietoris-Rips filtration of nuclei centers (Mischaikow and Nanda, 2013). When its performance was compared to a standard distance weighted discrimination classifier (Marron and Todd, 2007), nearly a four times improvement in classification accuracy was noted. Furthermore, for certain combinations of feature weightings, it was shown that topological features provide complementary information to patch based image appearance features. By using such topological features, they solve/address two main challenges in obtaining accurate cellular architectural characterization: the heterogeneity of spatial arrangements, both among patients and within single tumor samples, and differences in stain intensity which require manually determined phenotypic thresholds (Engers, 2007; Truesdale et al., 2011; Goodman et al., 2012; Helpap et al., 2012; Truong et al., 2013; Epstein et al., 2016; Evans et al., 2016). This improves performance over existing standard classifiers, which are more sensitive to noise, cannot model stain concentration variations, and have issues with larger cell arrangements (Aukerman et al., 2020).

In another paper, researchers used TDA to cluster prostate cancer histology into architectural groups consistent with the continuum of Gleason patterns, the most widely accepted system for evaluating prostate cancer architecture (Humphrey, 2004; Lawson et al., 2019). Persistent homology was used to compute persistence intensity diagrams (of zero and one dimensional components) of purely graded prostate cancer histopathology images of Gleason patterns 3–5. This revealed key insights into characteristics such as nuclei density, glandular shape, and inter-glandular arrangement. Furthermore, persistent homology was able to cluster these images into architectural groups through a rank descending persistence vector–the six resulting clusters provided a stable architectural continuum from well differentiated to poorly differentiated adenocarcinoma at an even finer level than the standard Gleason scale.

Persistent homology has also been used to characterize the spatial arrangement of immune and epithelial (tumor) cells within the breast cancer immune microenvironment from quantitative multiplex immunofluorescence (qmIF) imaging (Aukerman et al., 2020). Stain intensities and spatial coordinates of individual cells were collected from qmIF through nuclear segmentation, cytoplasmic definition, and stain quantification. In order to incorporate these stain intensities, instead of directly using a Rips or Cech filtration on the point cloud data (Chazal et al., 2009), a discretization process was first implemented to convert the point cloud data with stain intensity values into an image. Then, persistence diagrams were created from these images by using the opposite of the pixel stain intensity as the filter function. These diagrams were assessed as potential biomarkers of cancer subtype and prognostic biomarkers of overall survival using kernel mean embeddings (Gretton et al., 2012) with the sliced Wasserstein kernel (Carrière et al., 2017) and were shown to outperform the standard nearest neighbor analysis with a standard Gaussian kernel. Furthermore, a correlation analysis using constrained covariance (Herbrich et al., 2005) showed that the correlation between nearest neighbor and persistence diagrams were always <0.1, implying the features are nearly statistically independent and thus complementary.

## 7. Discussion

As we have seen in this paper, TDA has proven to be a powerful tool, yielding critical insights in the treatment prognosis, tumor segmentation and diagnosis, disease classification, and cellular architecture of cancer. But despite the many recent successes of TDA in the field of oncology, it is still a nascent field with much fruitful work yet to be done. Experimentally, to biologically validate the TDA methodology and results, it would be worth performing thorough studies to assess whether geometric and topological connectivity implies functional connectivity. Computationally, one area which deserves further exploration is the use of TDA to analyze time-series data (Ravishanker and Chen, 2019) in cancer. This has been done extensively in several other fields including climate analysis (Berwald et al., 2014), tracking stability of dynamical systems (Khasawneh and Munch, 2016), clustering populations of Tribolium flour beetles (Pereira and de Mello, 2015), analyzing motion sensor data during sports activities (Stolz et al., 2017), and financial time series data (Gidea, 2017; Truong, 2017; Gidea and Katz, 2018; Gidea et al., 2020). Though time series oncological data have been analyzed with varying degrees of success (Aoto et al., 2018; Kourou et al., 2020), TDA techniques of any sort have yet to be applied. Applying persistent homology techniques to time series microarray, cell anatomy imaging, or gene/pathway expression data, for example, may further help in disease classification, identifying intra-tumoral selection events, and contribute to a greater understanding of tumorigenesis. Another possible avenue of research is to investigate the process of angiogenesis, an inherently geometric and spatially dependent process, using persistent homology techniques. Specifically, we anticipate that TDA will help us understand the changes that occur in tumor vasculature morphology during cancer progression and under treatments. More importantly, we hope that connections between cancer vessel network and treatment prognosis can be found, such as by testing vessel normalization theory (Jain, 2005). In addition to the ideas presented above, it is worth noting that research into the use of TDA in oncology is sparse and, as such, there is much important and clinically relevant work to be done in simply applying well-understood persistent homology algorithms to broader classes of cancer data sets (note that most TDA analyses have been concentrated in just melanoma, brain, breast, and colorectal cancers) and in performing longitudinal studies across several cancer types.

## Author Contributions

AB conceptualized the project and wrote the sections 3–7. AB and ID wrote the section 1. ID wrote the section 2. NA wrote the sections 1, 3, 4, and 5. All authors contributed to the article and approved the submitted version.

## Conflict of Interest

The authors declare that the research was conducted in the absence of any commercial or financial relationships that could be construed as a potential conflict of interest.

## Footnotes

^1^While the intuitive definition will suffice for this paper, we have left out a number of details. For example if we use addition with ℤ_2_ coefficients, we can detect the Klein bottle surface (β_2_ = 1), while if we use ℤ coefficients, β_2_ = 0 since the Klein bottle does not bound a void. For computational speed, ℤ_2_ coefficients are frequently used when computing persistent homology.

## References

[B1] Abousamra S. Fassler D. Hou L. Zhang Y. Gupta R. Kurc T.. (2020). “Weakly-supervised deep stain decomposition for multiplex IHC images,” in 2020 IEEE 17th International Symposium on Biomedical Imaging (ISBI), 481–485. 10.1109/ISBI45749.2020.9098652

[B2] Adams H. Chepushtanova S. Emerson T. Hanson E. Kirby M. Motta F.. (2015). Persistence images: a stable vector representation of persistent homology. J. Mach. Learn. Res. 18, 1–35. Available online at: http://jmlr.org/papers/v18/16-337.html

[B3] Adams H. Emerson T. Kirby M. Neville R. Peterson C. Shipman P.. (2017). Persistence images: a stable vector representation of persistent homology. J. Mach. Learn. Res. 18, 1–35. Available online at: http://jmlr.org/papers/v18/16-337.html

[B4] Aiello M. Cavaliere C. D'Albore A. Salvatore M. (2019). The challenges of diagnostic imaging in the era of big data. J. Clin. Med. 8:316. 10.3390/jcm8030316 30845692 PMC6463157

[B5] Al-Kofahi Y. Lassoued W. Lee W. Roysam B. (2010). Improved automatic detection and segmentation of cell nuclei in histopathology images. IEEE Trans. Bio-Med. Eng. 57, 841–852. 10.1109/TBME.2009.2035102 19884070

[B6] Almendro V. Cheng Y. K. Randles A. Itzkovitz S. Marusyk A. Ametller E.. (2014). Inference of tumor evolution during chemotherapy by computational modeling and in situ analysis of genetic and phenotypic cellular diversity. Cell Rep. 6, 514–527. 10.1016/j.celrep.2013.12.041 24462293 PMC3928845

[B7] Alyass A. Turcotte M. Meyre D. (2015). From big data analysis to personalized medicine for all: challenges and opportunities. BMC Med. Genomics 8:33. 10.1186/s12920-015-0108-y 26112054 PMC4482045

[B8] Andor N. Graham T. A. Jansen M. Xia L. C. Aktipis C. A. Petritsch C.. (2016). Pan-cancer analysis of the extent and consequences of intratumor heterogeneity. Nat. Med. 22, 105–113. 10.1038/nm.3984 26618723 PMC4830693

[B9] Aoto Y. Okumura K. Hachiya T. Hase S. Wakabayashi Y. Ishikawa F.. (2018). Time-series analysis of tumorigenesis in a murine skin carcinogenesis model. Sci. Rep. 8:12994. 10.1038/s41598-018-31349-x 30158594 PMC6115443

[B10] Aparicio S. Caldas C. (2013). The implications of clonal genome evolution for cancer medicine. N. Engl. J. Med. 368, 842–851. 10.1056/NEJMra1204892 23445095

[B11] Arsuaga J. Baas N. A. Daniel DeWoskin Mizuno H. Pankov A. Park C. (2012). Topological analysis of gene expression arrays identifies high risk molecular subtypes in breast cancer. Applicable Algebra in Engineering, Communications and Comput. 23, 3–15. 10.1007/s00200-012-0166-8

[B12] Aukerman A. Carriére M. Chen C. Gardner K. Rabadán R. Vanguri R. (2020). “Persistent homology based characterization of the breast cancer immune microenvironment: a feasibility study,” in 36th International Symposium on Computational Geometry, Vol. 11 (Dagstuhl), 1–11.

[B13] Bauer U. (2019). Ripser: efficient computation of Vietoris-Rips persistence barcodes. arXiv: 1908.02518v1.

[B14] Bauer U. Lesnick M. (2014). “Induced matchings of barcodes and the algebraic stability of persistence,” in Computational Geometry (SoCG'14) (New York, NY: ACM), 355–364. 10.1145/2582112.2582168

[B15] Bendich P. Marron J. S. Miller E. Pieloch A. Skwerer S. (2016). Persistent homology analysis of brain artery trees. Ann. Appl. Stat. 10, 198–218. 10.1214/15-AOAS886 27642379 PMC5026243

[B16] Bergstra J. Bardenet R. Bengio Y. Kégl B. (2011). Algorithms for hyper-parameter optimization. Adv. Neural Inform. Process. Syst. 24, 1–9. Available online at: https://proceedings.neurips.cc/paper/2011/file/86e8f7ab32cfd12577bc2619bc635690-Paper.pdf

[B17] Berwald J. J. Gidea M. Vejdemo-Johansson M. (2014). Automatic recognition and tagging of topologically different regimes in dynamical systems. Discont. Nonlin. Complex. 3, 413–426. 10.5890/DNC.2014.12.004

[B18] Boissonnat J.-D. Pritam S. (2020). “Edge collapse and persistence of flag complexes,” in 36th International Symposium on Computational Geometry (SoCG 2020), Vol. 164 of Leibniz International Proceedings in Informatics (LIPIcs), eds S. Cabello and D. Z. Chen (Dagstuhl: Schloss Dagstuhl-Leibniz-Zentrum für Informatik), 19:1–19:15.

[B19] Bubenik P. (2015). Statistical topological data analysis using persistence landscapes. J. Mach. Learn. Res. 16, 77–102. Available online at: http://jmlr.org/papers/v16/bubenik15a.html

[B20] Bubenik P. (2020). “The persistence landscape and some of its properties,” in Topological Data Analysis, eds N. Baas, G. Carlsson, G. Quick, M. Szymik, M. Thaule (Geiranger: Springer), 97–117. 10.1007/978-3-030-43408-3_4

[B21] Bubenik P. Hull M. Patel D. Whittle B. (2020). Persistent homology detects curvature. Inverse Probl. 36:025008. 10.1088/1361-6420/ab4ac0

[B22] Bubenik P. Scott J. A. (2014). Categorification of persistent homology. Discrete Comput. Geom. 51, 600–627. 10.1007/s00454-014-9573-x

[B23] Bukkuri A. (2020). Optimal control analysis of combined chemotherapy-immunotherapy treatment regimens in a PKPD cancer evolution model. Biomath 9, 1–12. 10.11145/j.biomath.2020.02.137

[B24] Camara P. G. Rosenbloom D. I. Emmett K. J. Levine A. J. Rabadan R. (2016). Topological data analysis generates high-resolution, genome-wide maps of human recombination. Cell Syst. 3, 83–94. 10.1016/j.cels.2016.05.008 27345159 PMC4965322

[B25] Carlsson G. (2009). Topology and data. Bull. Am. Math. Soc. 46, 255–308. 10.1090/S0273-0979-09-01249-X

[B26] Carlsson G. Zomorodian A. Collins A. Guibas L. J. (2005). Persistence barcodes for shapes. Int. J. Shape Model. 11, 149–187. 10.1142/S0218654305000761

[B27] Carriére M. Cuturi M. Oudot S. (2017). “Sliced Wasserstein kernel for persistence diagrams,” in Proceedings of Machine Learning Research (Sydney, NSW).

[B28] Chazal F. Cohen-Steiner D. Glisse M. Guibas L. Oudot S. (2009). “Proximity of persistence modules and their diagrams,” in Proceedings of the Twenty-Fifth Annual Symposium on Computational Geometry (Aarhus: ACM), 237–246. 10.1145/1542362.1542407

[B29] Chazal F. de Silva V. Glisse M. Oudot S. (2016). The Structure and Stability of Persistence Modules. SpringerBriefs in Mathematics. Cham: Springer. 10.1007/978-3-319-42545-0_2

[B30] Chazal F. Fasy B. Lecci F. Michel B. Rinaldo A. Wasserman L. (2017). Robust topological inference: distance to a measure and kernel distance. J. Mach. Learn. Res. 18:40. Available online at: http://jmlr.org/papers/v18/15-484.html

[B31] Chittajallu D. R. Siekierski N. Lee S. Gerber S. Beezley J. Manthey D.. (2018). “Vectorized persistent homology representations for characterizing glandular architecture in histology images,” in 2018 IEEE 15th International Symposium on Biomedical Imaging (Washington, DC). 10.1109/ISBI.2018.8363562

[B32] Chung Y.-M. Hu C.-S. Lawson A. Smyth C. (2018). “Topological approaches to skin disease image analysis,” in IEEE International Conference on Big Data (Big Data) (Seattle, WA), 100–105. 10.1109/BigData.2018.8622175

[B33] Chung Y.-M. Lawson A. (2019). Persistence curves: a canonical framework for summarizing persistence diagrams. arXiv: 1904.07768.

[B34] Climent J. Dimitrow P. Fridlyand J. Palacios J. Siebert R. Albertson D. G.. (2007). Deletion of chromosome 11q predicts response to anthracycline-based chemotherapy in early breast cancer. Cancer Res. 67, 818–826. 10.1158/0008-5472.CAN-06-3307 17234794

[B35] Cohen-Steiner D. Edelsbrunner H. Harer J. (2007). Stability of persistence diagrams. Discrete Comput. Geom. 37, 103–120. 10.1007/s00454-006-1276-5

[B36] Crawford L. Monod A. Chen A. X. Mukherjee S. Rabadán R. (2020). Predicting clinical outcomes in glioblastoma: an application of topological and functional data analysis. J. Am. Stat. Assoc. 115, 1139–1150. 10.1080/01621459.2019.1671198

[B37] De Silva V. Carlsson G. (2004). “Topological estimation using witness complexes,” in Eurographics Symposium on Point-Based Graphics (Zurich), 157–166. 10.2312/SPBG/SPBG04/157-166

[B38] Dequeant M.-L. Ahnert S. Edelsbrunner H. Fink T. M. Glynn E. F. Hattem G.. (2008). Comparison of pattern detection methods in microarray time series of the segmentation clock. PLoS ONE 3:e2856. 10.1371/journal.pone.0002856 18682743 PMC2481401

[B39] DeWoskin D. Climent J. Cruz-White I. Vazquez M. Park C. Arsuaga J. (2010). Applications of computational homology to the analysis of treatment response in breast cancer patients. Topol. Appl. 157, 157–164. 10.1016/j.topol.2009.04.036

[B40] Dilsizian S. E. Siegel E. L. (2014). Artificial intelligence in medicine and cardiac imaging: harnessing big data and advanced computing to provide personalized medical diagnosis and treatment. Curr. Cardiol. Rep. 16:441. 10.1007/s11886-013-0441-8 24338557

[B41] Dimitriou N. Arandjelović O. Caie P. D. (2019). Deep learning for whole slide image analysis: an overview. Front. Med. 6:264. 10.3389/fmed.2019.00264 31824952 PMC6882930

[B42] Doyle S. Agner S. Madabhushi A. Feldman M. Tomaszewski J. (2008). “Automated grading of breast cancer histopathology using spectral clustering with textural and architectural image features,” in 5th IEEE International Symposium on Biomedical Imaging: From Nano to Macro (Paris), 496–499. 10.1109/ISBI.2008.4541041

[B43] Edelsbrunner H. Harer J. (2010). Computational Topology: An Introduction. Providence, RI: American Mathematical Society. 10.1090/mbk/069

[B44] Edelsbrunner H. Letscher D. Zomorodian A. (2002). Topological persistence and simplification. Discr. Comput. Geom. 28, 511–533. 10.1007/s00454-002-2885-2

[B45] Emmett K. Schweinhart B. Rabadan R. (2016). “Multiscale topology of chromatin folding,” in Proceedings of the 9th EAI Conference on Bio-inspired Information and Communications Technologies (New York, NY), 177–180. 10.4108/eai.3-12-2015.2262453

[B46] Engers R. (2007). Reproducibility and reliability of tumor grading in urological neoplasms. World J. Urol. 25, 595–605. 10.1007/s00345-007-0209-0 17828603

[B47] Epstein J. I. Zelefsky M. J. Sjoberg D. D. Nelson J. B. Egevad L. Magi-Galluzzi C.. (2016). A contemporary prostate cancer grading system: a validated alternative to the Gleason score. Eur. Urol. 69, 428–435. 10.1016/j.eururo.2015.06.046 26166626 PMC5002992

[B48] Evans S. M. Patabendi Bandarage V. Kronborg C. Earnest A. Millar J. Clouston D. (2016). Gleason group concordance between biopsy and radical prostatectomy specimens: a cohort study from Prostate Cancer Outcome Registry-Victoria. Prost. Int. 4, 145–151. 10.1016/j.prnil.2016.07.004 27995114 PMC5153432

[B49] Fass L. (2008). Imaging and cancer: a review. Mol. Oncol. 2, 115–152. 10.1016/j.molonc.2008.04.001 19383333 PMC5527766

[B50] Ferracin M. Pedriali M. Veronese A. Zagatti B. Gafá R. Magri E.. (2011). MicroRNA profiling for the identification of cancers with unknown primary tissue-of-origin. J. Pathol. 225, 43–53. 10.1002/path.2915 21630269 PMC4325368

[B51] Freije W. A. Castro-Vargas F. E. Fang Z. Horvath S. Cloughesy T. Liau L. M.. (2004). Gene expression profiling of gliomas strongly predicts survival. Cancer Res. 64, 6503–6510. 10.1158/0008-5472.CAN-04-0452 15374961

[B52] Garside K. Henderson R. Makarenko I. Masoller C. (2019). Topological data analysis of high resolution diabetic retinopathy images. PLoS ONE 14:e217413. 10.1371/journal.pone.0217413 31125372 PMC6534291

[B53] Ghrist R. (2008). Barcodes: the persistent topology of data. Bull. Am. Math. Soc. 45, 61–75. 10.1090/S0273-0979-07-01191-3

[B54] Ghrist R. W. (2014). Elementary Applied Topology, Vol. 1. Createspace Seattle.

[B55] Gidea M. (2017). “Topology data analysis of critical transitions in financial networks,” in 3rd International Winter School and Conference on Network Science (Tel Aviv), 47–59. 10.1007/978-3-319-55471-6_5

[B56] Gidea M. Goldsmith D. Katz Y. Roldan P. Shmalo Y. (2020). Topological recognition of critical transitions in time series of cryptocurrencies. Phys. A 548:123843. 10.1016/j.physa.2019.123843

[B57] Gidea M. Katz Y. (2018). Topological data analysis of financial time series: landscapes of crashes. Phys. A 491, 820–834. 10.1016/j.physa.2017.09.028

[B58] Goodman M. Ward K. C. Osunkoya A. O. Datta M. W. Luthringer D. Young A. N.. (2012). Frequency and determinants of disagreement and error in gleason scores: a population-based study of prostate cancer. Prostate 72, 1389–1398. 10.1002/pros.22484 22228120 PMC3339279

[B59] Gretton A. Borgwardt K. M. Rasch M. J. Smola A. Schölkopf B. Smola Gretton A. (2012). A kernel two-sample test. J. Mach. Learn. Res. 13, 723–773. Available online at: http://jmlr.org/papers/v13/gretton12a.html

[B60] Gu J. Taylor C. R. (2014). Practicing pathology in the era of big data and personalized medicine. Appl. Immunohistochem. Mol. Morphol. 22, 1–9. 10.1097/PAI.0000000000000022 24326463 PMC4206549

[B61] Guan Y. Stephens M. (2011). Bayesian variable selection regression for genome-wide association studies and other large-scale problems. Ann. Appl. Stat. 5, 1780–1815. 10.1214/11-AOAS455

[B62] Han W. Han M. R. Kang J. J. Bae J. Y. Lee J. H. Bae Y. J.. (2006). Genomic alterations identified by array comparative genomic hybridization as prognostic markers in tamoxifen-treated estrogen receptor-positive breast cancer. BMC Cancer 6:92. 10.1186/1471-2407-6-92 16608533 PMC1459182

[B63] Hanahan D. Weinberg R. A. (2000). The Hallmarks of Cancer. Technical report. 100, 57–70. 10.1016/S0092-8674(00)81683-9 10647931

[B64] Hatcher A. (2002). Algebraic Topology. Cambridge: Cambridge University Press.

[B65] Helpap B. Kristiansen G. Beer M. Köllermann J. Oehler U. Pogrebniak A.. (2012). Improving the reproducibility of the gleason scores in small foci of prostate cancer - Suggestion of diagnostic criteria for glandular fusion. Pathol. Oncol. Res. 18, 615–621. 10.1007/s12253-011-9484-6 22179685

[B66] Herbrich R. Smola A. Bousquet O. Schölkopf Bernhardschoelkopf B. Gretton A. Schölkopf Gretton B. (2005). Kernel methods for measuring independence. J. Mach. Learn. Res. 6, 2075–2129. Available online at: http://jmlr.org/papers/v6/gretton05a.html

[B67] Hong B.-W. Brady M. (2003). “A topographic representation for mammogram segmentation,” in International Conference on Medical Image Computing and Computer-Assisted Intervention (Montreal, QC), 730–737. 10.1007/978-3-540-39903-2_89

[B68] Horak D. Maletić S. Rajković M. (2009). Persistent homology of complex networks. J. Stat. Mech. 2009:P03034. 10.1088/1742-5468/2009/03/P03034

[B69] Horlings H. M. Lai C. Nuyten D. S. Halfwerk H. Kristel P. Van Beers E.. (2010). Integration of DNA copy number alterations and prognostic gene expression signatures in breast cancer patients. Clin. Cancer Res. 16, 651–663. 10.1158/1078-0432.CCR-09-0709 20068109

[B70] Humphrey P. A. (2004). Gleason grading and prognostic factors in carcinoma of the prostate. Modern Pathol. 17, 292–306. 10.1038/modpathol.3800054 14976540

[B71] Ishwaran H. Rao J. S. (2005). Spike and slab variable selection: frequentist and bayesian strategies. Ann. Stat. 33, 730–773. 10.1214/009053604000001147

[B72] Jain R. K. (2005). Normalization of tumor vasculature: an emerging concept in antiangiogenic therapy. Sci. Rev. 307, 58–62. 10.1126/science.1104819 15637262

[B73] Khan A. El-Daly H. Simmons E. Rajpoot N. (2013). HyMaP: A hybrid magnitude-phase approach to unsupervised segmentation of tumor areas in breast cancer histology images. J. Pathol. Inform. 4(Suppl):S1. 10.4103/2153-3539.109802 23766931 PMC3678741

[B74] Khasawneh F. A. Munch E. (2016). Chatter detection in turning using persistent homology. Mech. Syst. Signal Process. 70–71, 527–541. 10.1016/j.ymssp.2015.09.046

[B75] Kimura M. Obayashi I. Takeichi Y. Murao R. Hiraoka Y. (2018). Non-empirical identification of trigger sites in heterogeneous processes using persistent homology. Sci. Rep. 8:3553. 10.1038/s41598-018-21867-z 29476108 PMC5824834

[B76] Kourou K. Rigas G. Papaloukas C. Mitsis M. Fotiadis D. I. (2020). Cancer classification from time series microarray data through regulatory Dynamic Bayesian Networks. Comput. Biol. Med. 116:103577. 10.1016/j.compbiomed.2019.103577 32001012

[B77] Kusano G. Hiraoka Y. Fukumizu K. (2016). “Persistence weighted Gaussian kernel for topological data analysis,” in International Conference on Machine Learning (New York, NY), 2004–2013.

[B78] Laurie C. C. Laurie C. A. Rice K. Doheny K. F. Zelnick L. R. McHugh C. P.. (2012). Detectable clonal mosaicism from birth to old age and its relationship to cancer. Nat. Genet. 44, 642–650. 10.1038/ng.2271 22561516 PMC3366033

[B79] Lawson P. Sholl A. B. Brown J. Q. Fasy B. T. Wenk C. (2019). Persistent homology for the quantitative evaluation of architectural features in prostate cancer histology. Sci. Rep. 9:1139. 10.1038/s41598-018-36798-y 30718811 PMC6361896

[B80] Li C. H. Tam P. K. (1998). An iterative algorithm for minimum cross entropy thresholding. Pattern Recogn. Lett. 19, 771–776. 10.1016/S0167-8655(98)00057-9

[B81] Li M. An H. Angelovici R. Bagaza C. Batushansky A. Clark L. Coneva V.. (2018). Topological data analysis as a morphometric method: Using persistent homology to demarcate a leaf morphospace. Front. Plant Sci. 9:553. 10.3389/fpls.2018.00553 29922307 PMC5996898

[B82] Lockwood S. Krishnamoorthy B. (2015). “Topological features in cancer gene expression data,” in Pacific Symposium on Biocomputing (Kohala Coast). 10.1142/9789814644730_0012 25592573

[B83] Macenko M. Niethammer M. Marron J. Borland D. Woosley J. T. Guan X. (2009). “A method for normalizing histology slides for quantitative analysis,” in IEEE International Symposium on Biomedical Imaging: From Nano to Macro (Boston, MA), 1107–1110. 10.1109/ISBI.2009.5193250

[B84] Maley C. C. Galipeau P. C. Finley J. C. Wongsurawat V. J. Li X. Sanchez C. A.. (2006). Genetic clonal diversity predicts progression to esophageal adenocarcinoma. Nat. Genet. 38, 468–473. 10.1038/ng1768 16565718

[B85] Maley C. C. Reid B. J. (2005). Natural selection in neoplastic progression of Barrett's esophagus. Semin. Cancer Biol. 15, 474–483. 10.1016/j.semcancer.2005.06.004 16043360

[B86] Marquard A. M. Birkbak N. J. Thomas C. E. Favero F. Krzystanek M. Lefebvre C.. (2015). TumorTracer: a method to identify the tissue of origin from the somatic mutations of a tumor specimen. BMC Med. Genomics 8:58. 10.1186/s12920-015-0130-0 26429708 PMC4590711

[B87] Marron J. S. Todd M. (2007). Distance-weighted discrimination. J. Am. Stat. Assoc. 102, 1267–1271. 10.1198/016214507000001120 PMC2996856 21152360

[B88] Mileyko Y. Mukherjee S. Harer J. (2011). Probability measures on the space of persistence diagrams. Inverse Probl. 27:124007. 10.1088/0266-5611/27/12/124007

[B89] Mischaikow K. Nanda V. (2013). Morse theory for filtrations and efficient computation of persistent homology. Discr. Comput. Geom. 50, 330–353. 10.1007/s00454-013-9529-6

[B90] Moran S. Martínez-Cardús A. Sayols S. Musulén E. Bala ná C. Estival-Gonzalez A.. (2016). Epigenetic profiling to classify cancer of unknown primary: a multicentre, retrospective analysis. Lancet Oncol. 17, 1386–1395. 10.1016/S1470-2045(16)30297-2 27575023

[B91] Munkres J. R. (1984). Elements of Algebraic Topology. Menlo Park, CA: Addison-Wesley Publishing Company.

[B92] Neve R. M. Chin K. Fridlyand J. Yeh J. Baehner F. L. Fevr T.. (2006). A collection of breast cancer cell lines for the study of functionally distinct cancer subtypes. Cancer Cell 10, 515–527. 10.1016/j.ccr.2006.10.008 17157791 PMC2730521

[B93] Nicolau M. Levine A. J. Carlsson G. (2011). Topology based data analysis identifies a subgroup of breast cancers with a unique mutational profile and excellent survival. Proc. Natl. Acad. Sci. U.S.A. 108, 7265–7270. 10.1073/pnas.1102826108 21482760 PMC3084136

[B94] Nicolau M. Tibshirani R. Børresen-Dale A. L. Jeffrey S. S. (2007). Disease-specific genomic analysis: identifying the signature of pathologic biology. Bioinformatics 23, 957–965. 10.1093/bioinformatics/btm033 17277331

[B95] Nielson J. L. Cooper S. R. Yue J. K. Sorani M. D. Inoue T. Yuh E. L.. (2017). Uncovering precision phenotype-biomarker associations in traumatic brain injury using topological data analysis. PLoS ONE 12:e169490. 10.1371/journal.pone.0169490 28257413 PMC5336356

[B96] Nutt C. L. Mani D. R. Betensky R. A. Tamayo P. Cairncross J. G. Ladd C.. (2003). Gene expression-based classification of malignant gliomas correlates better with survival than histological classification. Cancer Res. 63, 1602–1607. 12670911

[B97] Obayashi I. Hiraoka Y. (2018). Persistence diagrams with linear machine learning models. J. Appl. Comput. Topol. 1, 421–449. 10.1007/s41468-018-0013-5

[B98] Otter N. Porter M. A. Tillmann U. Grindrod P. Harrington H. A. (2017). A roadmap for the computation of persistent homology. EPJ Data Sci. 6:17. 10.1140/epjds/s13688-017-0109-5 32025466 PMC6979512

[B99] Oudot S. Y. (2015). Persistence Theory: From Quiver Representations to Data Analysis, Vol. 209 of Mathematical Surveys and Monographs. Providence, RI: American Mathematical Society. 10.1090/surv/209

[B100] Oyama A. Hiraoka Y. Obayashi I. Saikawa Y. Furui S. Shiraishi K.. (2019). Hepatic tumor classification using texture and topology analysis of non-contrast-enhanced three-dimensional T1-weighted MR images with a radiomics approach. Sci. Rep. 9:8764. 10.1038/s41598-019-45283-z 31217445 PMC6584736

[B101] Pereira C. M. de Mello R. F. (2015). Persistent homology for time series and spatial data clustering. Expert Syst. Appl. 42, 6026–6038. 10.1016/j.eswa.2015.04.010

[B102] Phillips H. S. Kharbanda S. Chen R. Forrest W. F. Soriano R. H. Wu T. D.. (2006). Molecular subclasses of high-grade glioma predict prognosis, delineate a pattern of disease progression, and resemble stages in neurogenesis. Cancer Cell 9, 157–173. 10.1016/j.ccr.2006.02.019 16530701

[B103] Qaiser T. Sirinukunwattana K. Nakane K. Tsang Y. W. Epstein D. Rajpoot N. (2016). “Persistent homology for fast tumor segmentation in whole slide histology images,” in Procedia Computer Science, Vol. 90 (Loughborough: Elsevier B.V.), 119–124. 10.1016/j.procs.2016.07.033

[B104] Rabadán R. Mohamedi Y. Rubin U. Chu T. Alghalith A. N. Elliott O.. (2020). Identification of relevant genetic alterations in cancer using topological data analysis. Nat. Commun. 11, 1–10. 10.1101/2020.01.30.922310 32732999 PMC7393176

[B105] Ravishanker N. Chen R. (2019). Topological data analysis (TDA) for time series. arXiv: 1909.10604.

[B106] Reinhard E. Ashikhmin M. Gooch B. Shirley P. (2001). Color transfer between images. IEEE Comput. Graph. Appl. 21, 34–41. 10.1109/38.946629

[B107] Reininghaus J. Huber S. Bauer U. Kwitt R. (2015). “A stable multi-scale kernel for topological machine learning,” in Proceedings of the IEEE Conference on Computer Vision and Pattern Recognition (Boston, MA), 4741–4748. 10.1109/CVPR.2015.7299106

[B108] Reuter J. A. Spacek D. V. Snyder M. P. (2015). High-throughput sequencing technologies. Mol. Cell 58, 586–597. 10.1016/j.molcel.2015.05.004 26000844 PMC4494749

[B109] Roychowdhury S. Iyer M. K. Robinson D. R. Lonigro R. J. Wu Y. M. Cao X.. (2011). Personalized oncology through integrative high-throughput sequencing: a pilot study. Sci. Transl. Med. 3, 1–12. 10.1126/scitranslmed.3003161 22133722 PMC3476478

[B110] Rucco M. Merelli E. Herman D. Ramanan D. Petrossian T. Falsetti L.. (2015). Using Topological Data Analysis for diagnosis pulmonary embolism. arXiv:1409.5020v1. 9, 41–55.26515513

[B111] Seemann L. Shulman J. Gunaratne G. H. (2012). A robust topology-based algorithm for gene expression profiling. ISRN Bioinform. 2012:381023. 10.5402/2012/381023 25969748 PMC4393071

[B112] Siddiqui S. Shikotra A. Richardson M. Doran E. Choy D. Bell A.. (2018). Airway pathological heterogeneity in asthma: visualization of disease microclusters using topological data analysis. J. Aller. Clin. Immunol. 142, 1457–1468. 10.1016/j.jaci.2017.12.982 29550052

[B113] Singh G. Mémoli F. Carlsson G. (2007). “Topological methods for the analysis of high dimensional data sets and 3D object recognition,” in Eurographics Symposium on Point-Based Graphics (Prague).

[B114] Singh N. Couture H. D. Marron J. S. Perou C. Niethammer M. (2014). “Topological descriptors of histology images,” in Machine Learning in Medical Imaging (Boston, MA). 10.1007/978-3-319-10581-9_29

[B115] Søndergaard D. Nielsen S. Pedersen C. N. Besenbacher S. (2017). Prediction of primary tumors in cancers of unknown primary. J. Integr. Bioinform. 14:20170013. 10.1515/jib-2017-0013 28686574 PMC6042823

[B116] Stack E. C. Wang C. Roman K. A. Hoyt C. C. (2014). Multiplexed immunohistochemistry, imaging, and quantitation: a review, with an assessment of Tyramide signal amplification, multispectral imaging and multiplex analysis. Methods 70, 46–58. 10.1016/j.ymeth.2014.08.016 25242720

[B117] Stolz B. J. Harrington H. A. Porter M. A. (2017). Persistent homology of time-dependent functional networks constructed from coupled time series. Chaos 27:047410. 10.1063/1.4978997 28456167

[B118] Suwinski P. Ong C. K. Ling M. H. Poh Y. M. Khan A. M. Ong H. S. (2019). Advancing personalized medicine through the application of whole exome sequencing and big data analytics. Front. Genet. 10:49. 10.3389/fgene.2019.00049 30809243 PMC6379253

[B119] Tahmassebi A. Schulte M. H. Gandomi A. H. Goudriaan A. E. McCann I. Meyer-Baese A. (2018). “Deep learning in medical imaging: FMRI big data analysis via convolutional neural networks,” in ACM International Conference Proceeding Series (Pittsburgh, PA: Association for Computing Machinery), 1–4. 10.1145/3219104.3229250

[B120] Truesdale M. D. Cheetham P. J. Turk A. T. Sartori S. Hruby G. W. Dinneen E. P.. (2011). Gleason score concordance on biopsy-confirmed prostate cancer: is pathological re-evaluation necessary prior to radical prostatectomy? BJU Int. 107, 749–754. 10.1111/j.1464-410X.2010.09570.x 20840549

[B121] Truong M. Slezak J. A. Lin C. P. Iremashvili V. Sado M. Razmaria A. A.. (2013). Development and multi-institutional validation of an upgrading risk tool for Gleason 6 prostate cancer. Cancer 119, 3992–4002. 10.1002/cncr.28303 24006289 PMC4880351

[B122] Truong P. (2017). An exploration of topological properties of high-frequency onedimensional financial time series data using TDA (Ph.D. thesis). KTH Royal Institute of Technology, Stockholm, Sweden.

[B123] Tschandl P. Rosendahl C. Kittler H. (2018). Data descriptor: the HAM10000 dataset, a large collection of multi-source dermatoscopic images of common pigmented skin lesions. Sci. Data 5:180161. 10.1038/sdata.2018.161 30106392 PMC6091241

[B124] Turner K. Mukherjee S. Boyer D. M. (2014). Persistent homology transform for modeling shapes and surfaces. Inf. Inference 3, 310–344. 10.1093/imaiai/iau011

[B125] Verhaak R. G. Hoadley K. A. Purdom E. Wang V. Qi Y. Wilkerson M. D.. (2010). Integrated genomic analysis identifies clinically relevant subtypes of glioblastoma characterized by abnormalities in PDGFRA, IDH1, EGFR, and NF1. Cancer Cell 17, 98–110. 10.1016/j.ccr.2009.12.020 20129251 PMC2818769

[B126] Vikeså J. Møller A. K. Kaczkowski B. Borup R. Winther O. Henao R.. (2015). Cancers of unknown primary origin (CUP) are characterized by chromosomal instability (CIN) compared to metastasis of know origin. BMC Cancer 15:151. 10.1186/s12885-015-1128-x 25885340 PMC4404593

[B127] Wadhwa R. R. Williamson D. F. K. Dhawan A. Scott J. G. (2018). TDAstats: R pipeline for computing persistent homology in topological data analysis. J. Open Source Softw. 3:860. 10.21105/joss.00860 33381678 PMC7771879

[B128] Wang G. (2016). A perspective on deep imaging. IEEE Access 4, 8914–8924. 10.1109/ACCESS.2016.2624938

[B129] Weinberger S. (2014). The complexity of some topological inference problems. Found. Comput. Math. 14, 1277–1285. 10.1007/s10208-013-9152-1

[B130] Wilkerson A. C. Chintakunta H. Krim H. (2014). “Computing persistent features in big data: a distributed dimension reduction approach,” in 2014 IEEE International Conference on Acoustics, Speech and Signal Processing (ICASSP) (Florence), 11–15. 10.1109/ICASSP.2014.6853548

[B131] Xiaohua C. Brady M. Rueckert D. (2004). “Simultaneous segmentation and registration for medical image,” in International Conference on Medical Image Computing and Computer-Assisted Intervention (Saint-Malo), 663–670. 10.1007/978-3-540-30135-6_81

[B132] Yu K. H. Zhang C. Berry G. J. Altman R. B. Ré C. Rubin D. L.. (2016). Predicting non-small cell lung cancer prognosis by fully automated microscopic pathology image features. Nat. Commun. 7, 1–10. 10.1038/ncomms12474 27527408 PMC4990706

[B133] Yuan Y. Failmezger H. Rueda O. M. Raza Ali H. Gräf S. Chin S. F.. (2012). Quantitative image analysis of cellular heterogeneity in breast tumors complements genomic profiling. Sci. Transl. Med. 4:157ra143. 10.1126/scitranslmed.3004330 23100629

[B134] Zhang Y. Brady M. Smith S. (2001). Segmentation of brain MR images through a hidden markov random field model and the expectation-maximization algorithm. IEEE Trans. Med. Imaging 20:45. 10.1109/42.906424 11293691

[B135] Zhao B. Pritchard J. R. Lauffenburger D. A. Hemann M. T. (2014). Addressing genetic tumor heterogeneity through computationally predictive combination therapy. Cancer Discov. 4, 166–174. 10.1158/2159-8290.CD-13-0465 24318931 PMC3975231

[B136] Zhou X. Carbonetto P. Stephens M. (2013). Polygenic modeling with bayesian sparse linear mixed models. PLoS Genet. 9:e1003264. 10.1371/journal.pgen.1003264 23408905 PMC3567190

[B137] Zomorodian A. (2010). “The tidy set: a minimal simplicial set for computing homology of clique complexes [extended abstract],” in Computational Geometry (SCG'10) (New York, NY: ACM), 257–266. 10.1145/1810959.1811004

[B138] Zomorodian A. Carlsson G. (2005). Computing persistent homology. Discrete Comput. Geom. 33, 249–274. 10.1007/s00454-004-1146-y

